# Dr. Maclean, on Digitalis

**Published:** 1800-08

**Authors:** 


### *To the Editors of the Medical and Phyſical Journal.*

Gentlemen,

In my communication for February laſt, I announced an intention of coming forward in the ſpring with an 'Eſſay on the general Properties of the Fox-glove.' I pledged myſelf likewiſe to notice ſome Strictures of Dr. Drake on a letter I addreſſed to you in Auguſt laſt. The preſſure of various avocations, however, have precluded me from fulfilling my engagements; but juſtice to myſelf, and to the cauſe I have eſpouſed, require I ſhould, without farther delay, perform the latter part of my intention.

When I firſt volunteered my ſervices on this important ſubject, I did ſo, as I imagined, with the view of ſupporting the credit and real character of my favourite plant. I had for ſome years reared it with attention in my garden, and brought it to a degree of perfection which I had never obſerved it to attain in the natural ſtate. My experience of its virtues in various diſeaſes was extenſive; and my ſucceſs in hydrothorax by means of a combination of this herb, with certain other means, (which ſhall in due time be laid before the public) was great; I had reaſon to believe, beyond any former example. I had recently uſed it in ſome caſes, (about twenty,) of conſumption; therefore when its merits, in this laſt diſeaſe, began to be publicly diſcuſſed, I fancied myſelf in ſome degree prepared to bear a part in the diſcuſſion; at leaſt, by adding my ſtock of facts and obſervations to the maſs of evidence already before the public.

But, in candidly and faithfully detailing the reſults of the trials I had made, I was concerned to find I muſt neceſſarily differ in many important points from the reſpectable authors who had recently gone before me. They held out the moſt ſanguine hopes of ſucceſs, and ſeemed already to congratulate themſelves, that an effectual remedy was at length diſcovered for that cruel ſcourge of mankind, conſumption; while I endeavoured to prepare the public mind for more moderate expectations. Moſt earneſtly do I wiſh their hopes had been realiſed, while mine proved otherwiſe. But, unfortunately for the human race, the reverſe has been the event; ſubſequent experience having fully confirmed the truth of what I then advanced.

It is proper to obſerve, however, that Dr. Beddoes, one of the authors alluded to, with a liberality characteriſtic of great minds, finding he had exceeded due bounds in his encomiums comiums on the Fox-glove, evidently from the high colouring of his correfpondents, has qualified his original language in a fecond edition of his excellent work on Confumption; and it augurs rather unfavourably to fee his active mind again in fearch of a remedy for this ftill too generally fatal difeafe.

I obferve, likewife, the fentiments of the Monthly Reviewers, in their candid, judicious, and impartial analyfis of every thing that has recently been written on this plant, accord fully with mine. Not fo, however, with Dr. Drake; whatever I have faid on the fubject feems alike obnoxious to him: he not only objects to what I ventured to advance as facts, but reprefents me as difcouraging the ufe of the Fox-glove from groundlefs fears of its deftructive qualities. To him, therefore, permit me to addrefs a few lines, provided their infertion in your truly valuable Journal be not likely in any degree to injure its juftly-acquired reputation.

From the fortunate refults of the two cafes publifhed in the Contributions, &c. Dr. Drake thus concludes his encomium on the Fox-glove.

"I may, I think, without hefitation, affirm that the early exhibition of the *faturated* tincture in confumption will in general prove fuccefsful, and even when the difeafe is far advanced; provided the patient has but ftrength enough left to endure a gradual depreffion of the circulation, a refult equally fortunate may be expected. That this can be done even in circumftances of debility to an extent adequate to effect a cure, and without ficknefs, languor, or lofs of appetite, the cafes appended will fatisfactorily atteft." *(Med. Contr. p.* 487.*)*

The principal grounds of my diffent from this confident inference have already been ftated. But this language, though ftrong, fell far fhort of that which was induftrioufly promulgated by the Doctor's friends; and if my memory do not deceive me, by himfelf in a converfation we had twice on the fubject. It had fucceeded, it was urged, in every inftance in which it was tried; and the moft hopelefs cafes were incapable of refifting its fpecific powers. In vain did I affure them (his venerable predeceffor Dr. Gibbon among the number) they deceived themfelves; that its powers were limitted even in the early ftages of true confumption. In anfwer to this it was faid, that I had been ufing the powder, from which, it was contended, "the wifhed-for effect would feldom be produced." Every thing was to be effected by the wonder-working influence of the tincture: not even the tea of long life of St. Germain, the boafted elixir of Caglioftro, or the magnetic influence of Mefmer, were promifed to atchieve more. But figure my aftonifhment on finding all the cafes in which it was faid to have fucceeded

ſucceeded dwindle away to two! I had myſelf been ſucceſsful in more than double the number; and I thought it now incumbent upon me to come forward, as well to prepare the public mind for what my experience enabled me to ſay it was really capable of performing, as to ſtate ſome particulars reſpecting its mode of preparation, exhibition, doſes, and effects, which appeared to have been overlooked, or imperfectly underſtood, and conſequently to have retarded the more general interdiction of this valuable herb. That I have done this has been productive of no regret to me. If I may be allowed to judge from the many private, and not a few public teſtimonies of approbation beſtowed upon my remarks, although uſhered into the world in the aukward and ungraceful dreſs too generally incident to haſty productions, I have reaſon to believe they have been favourably received by all except by Dr. Drake. For, ſays he, "ſome apprehenſions are nevertheleſs entertained that injury may accrue to medicine, from the reports already publiſhed in favour of this plant. Your Journal for Auguſt contains an ingenious communication" (a communication which I would ſay ought to be burnt by the hands of the hangman, if the Doctor's objections were well founded,) "from Dr. Maclean, of Sudbury; in which, however, notwithſtanding his own teſtimony to the good effects of the digitalis in conſumption," he concludes a paragraph, and commences another, in the following words:" "*I ſee*, ſays he, "*the moſt ſerious evils begin already to reſult from its not anſwering the high expectations that have been raiſed.*"—"*Others begin to loſe their confidence in it from ſimilar failures*; *whereas*, *had it been brought forward with its true character ſtamped upon it*, *this would not be the caſe.*" (*Medical and Phyſical Journal*, *vol.* ii. *p.* 417). And afterwards, "I may alſo with confidence affirm that no evil of any magnitude can ariſe from the uſe of Digitalis in tubercular conſumption, if properly exhibited, and that he who ſhall haſten to employ it early in the diſeaſe, will, in proportion to his promptitude, be a benefactor to mankind." And farther, "I am likewiſe well convinced that miſchief may, and will, probably be produced by an injudicious and indiſcriminate uſe of this active plant, &c." (p. 418).

I am really not ſolicitous to refute the imputations thus directly and obliquely conveyed, becauſe I am perſuaded, every one who has peruſed my remarks, and I am certain all thoſe who are acquainted with my ſentiments of the Fox-glove, will readily acquit me of them. Were it poſſible to attach ſuch an interpretation to my words, the teſtimony of every practitioner within the circle of my regular rides, and many beyond it, with whom I have occaſional intercourſe, would poſitively contradict tradict it. For, whatever be the fate of this plant ultimately, the merit or demerit of bringing it into early and general use in this part of the country, I claim as attaching exclusively to me. I mean not, however, to infer that it had not been used by others before me; many had tried it in dropsy: but from want of success, owing to the causes already mentioned, I found it generally deserted; for when I fixed my residence in Sudbury, I recollect only two medical men who were in the habit of occasionally resorting to it; whereas, at this time, it is used by all, and this from a conviction of its extraordinary powers. And if the Doctor's scepticism in this respect be not invincible, he might easily persuade himself that my garden has liberally supplied the greater number with genuine leaves, not inferior in any degree to those of East Bergholt, where he loaded his botanical cart, and I may add, to those of any in the kingdom. Of this more hereafter. But, while the Doctor expresses his surprise that I do not assent to his conclusions respecting the almost specific virtues of the plant, in direct contradiction to my own experience, and, I venture to predict, to that of every man of extensive practice in this kingdom, he seems to have unluckily forgotten that he himself has, by a regular but insensible gradation, fallen into my opinion. "Of five cases," observes he, "of confirmed consumption, (including those of Grimes and Morris,) three have been perfectly cured. I have no expectation, however, that upon a larger scale, the proportion of fortunate to fatal result, would be what my experience has given." *(ib. 268.)* And again, in his last communication, "The experience I have now had, indeed, thoroughly convinces me that the digitalis will be found adequate to the cure of several cases of tubercular consumption, though advanced into the second stage; that it will for a time suspend the progress of the symptoms in almost every instance; and that in catarrh, in hæmoptoe, and in vomica, it will in some measure approach to what is commonly implied by the term specific, &c." *(ib. 417).* How different is this from the general tenor of the first paragraph transcribed! Had such language been originally used, the objections urged by me to this part of his Essay, would never have existed, because it would have accorded with the sentiments delivered by me some months before, as may be seen in pages 115, 116, and 117 of the Medical and Physical Journal, vol. ii. to which I beg leave to refer the reader. He will perceive, that instead of saying any thing adverse to the use of the Fox-glove in consumption, I strenuously recommended it as a remedy which I trusted would be found by others as it had been by me, "the most efficacious that had hitherto been resorted to," although experience taught me, "its powers were limitted in the the early ftage." *(p.* 115.) Yet while I earneftly encouraged farther trials, I was folicitous to guard the unexperienced in its ufe againft the obvious confequences of fuch exaggerated encomiums. I faid, that I faw "the moft ferious evils begin already to refult from its not anfwering the high expectations that had been raifed." I fay fo ftill; and in all human concerns it muft neceffarily be fo. When a character has been ftamped upon any thing which experience contradicts, difappointment, often difguft, follows; and fuch is the force of prejudice, that it fometimes finks beneath its real value. This has unfortunately been the fate of the Digitalis with many, efpecially thofe whofe minds were impreffed with the belief, that "the exhibition of the faturated tincture would in general prove fuccefsful in confumption." But if the Doctor has at length (fome months after his firft Effay appeared) fallen into my views, why exprefs his furprife at my rejecting from my creed, what he himfelf no longer believes? It was not to what he intended to fay, write, or publifh, that my objections were urged, but to what he had actually faid, written, and publifhed. Although born, and my early days paffed in a country where the belief in the fecond fight was general, I had not yet learnt that happy gift of feeing through the impenetrable veil which conceals futurity.

That the Fox-glove has fallen into much greater difrepute in fome parts of this country, in confumption, than it really merits, not only from the caufes which excited alarm in me, but from the failures of the Doctor himfelf, is a fact too well known; and it is now no lefs difficult to convince fome of thofe who perfuaded themfelves that every cafe of confumption would give way to this medicine, that it will fucceed in any cafe, than it was before that it would not always fucceed.

In vomica too, as well as in confumption, "in which," fays the Doctor, "it will, in fome meafure, approach to what is commonly implied by the term fpecific; a word, however, which," he thinks, "fhould be banifhed from medicine;" if he had but patiently awaited the refults of a more enlarged experience, he would have been more humble in his expectations. In this I have done much good with the Fox-glove, probably more than it has yet fallen to his lot to do, unlefs he has a great deal in ftore; but it has fometimes failed me in cafes where I had reafon to be fanguine. In the cafe of a maiden lady, 41 years of age, it approached indeed to what might be termed a fpecific. Mifs Harrifon had a vomica fo extenfive as frequently to expectorate a quart of thick offenfive pus, fometimes tinged with blood, in twenty-four hours. It was, at one time, not only combined with ferous effufion in the cavities of the cheſt, but with general anaſarca, and her ſtrength ſo far exhauſted as to preclude all hope of recovery. But ſhe ſurvived this formidable attack, and ſeveral others for the laſt two years, by the aid of the Fox-glove. Her conſtitution has been ſo much ſhattered, however, by theſe repeated attacks, that, as might naturally be imagined, ſhe ſeldom enjoys what may be termed *good* health. She always has a phial of the tincture, or pills made of the powder, in her poſſeſſion, to which ſhe is frequently obliged to reſort, and invariably with relief. Other auxiliaries were not at the ſame time loſt ſight of.

"Now," continues Dr. Drake, "in the Contributions the Digitalis is related to have been given with the view to cure *tubercular conſumption*; and if by experience it has been found beneficial in hæmoptoe, and ſpaſmodic aſthma, does it follow, that ſhould it fail in the former complaint, it muſt neceſſarily fall into diſrepute in the latter diſeaſes? Surely Dr. M. ſhould have treated with contempt thoſe who are weak enough to think and argue in this manner." *(ib.* 417, *et ſeq.)* Dr. D. has unfortunately committed an overſight here. The caſes publiſhed by him were, he tells us, of *tubercular conſumption:* to theſe only did my remarks apply; hence, as he has been refuting objections created by himſelf, I hope he is ſatisfied with his own refutation; I have even poſitively affirmed that "Digitalis will be found highly beneficial in aſthmatic affections." *(ib. p.* 117, *vol.* ii.*)* Applying them, however, to conſumption, as they were done by me, I ſuſpect not only thoſe reſpectable practitioners he politely adviſes me "to treat with contempt for being weak enough, &c." but every one of experience who will candidly decide on the merits of the Fox-glove, would be diſpoſed to retort the charge of credulous weakneſs upon himſelf, for promiſing general ſucceſs from a remedy he had uſed only in two caſes! (in a diſeaſe too which had hitherto very generally reſiſted all the efforts of art;) if he did not fall under the imputation of a violation of the rules of good manners.

To his objections to the proportions of leaves to ſpirituous menſtrua given by me, and the ſtrength of the tinctures thus drawn, I have already ſpoken, *(ib. vol.* iii. *p.* 152*)*. And as he has been ſilent in his laſt reply, this point may, I preſume, be conſidered as decided. I ſhall beg only to obſerve, that the term *ſaturated*, on which he dwells with ſo much emphaſis, and to which, he ſays, tinctures made with leſs proportions than one ounce of the dried leaves to five of proof ſpirit, "can offer no claim," was never uſed by me, becauſe I conſidered it a vague and unmeaning term, as applied by Dr. Darwin and him, having aſcertained by repeated trials, that what was *ſaturated* in one temperature temperature of their tincture was not fo in another, from the great depofition which took place. But if by the term *faturated*, be underftood that property by which the fpirit retains *permanently* a given quantity of the active parts of the leaf, in every variety of temperature in which it is likely to be placed, (not that by which it does fo for a given time, and in a certain temperature only) the tinctures made agreeably to my formula, have, I think, a much ftronger claim to it.

By digeftion for a week or more, a very important object is gained; half the proportions of the leaf being fufficient; and a larger quantity of the tincture, of equal, more uniform, and permanent ftrength, being obtained.

For a digeftion of twenty-four hours, there is no precedent in the Pharmacopeia of the learned Colleges of London or Edinburgh; undoubtedly, becaufe experience taught them it was not fufficient time for the fpirit to extract and retain the active parts of vegetables. By their decifion, in regard to the formula fubmitted for their confideration, will I readily abide. I now conduct the digeftion in a cool temperature, by which I find the tincture is ftill lefs liable to variation.

Ample experience now warrants me in repeating what I formerly faid, (*ib.* p. 52) that I have not been able to exceed ninety drops in the day, except in the inftance mentioned. I find fixty drops in twenty-four hours in general fully adequate to place the ftrongeft habits under its influence, of which many examples fhall in due time be given.

A young woman, who was laft fummer cured of confumption, and is at this time in perfect health, was nearly poifoned by 66 drops in twenty-fours. She began with 120 drops in a half pint faline mixture, accurately meafured, of which two table fpoonsful were taken three times a day. The quantity of tincture was gradually increafed to 176 drops: Before fhe began the mixture which contained this quantity, her recovery was rapid, and the habit was fenfibly under the influence of the medicine. The firft few dofes occafioned ficknefs and vomiting, yet fhe perfifted until the whole was confumed. A female relation now came to me, to fay fhe was dying, having retched and vomited inceffantly for three days, and that fhe was now fo bad and fo faint, they could not keep life in her." By means of folid opium made into pills with confectio aromatica, (for every thing liquid was inftantly rejected) warm anodyne embrocations, and an anodyne glyfter, fhe was in a few days pronounced in fafety, and a fortnight more completed the cure. The patient being at a diftance from Mr. Rainbird, of Colne in Effex, (who attended with me,) and myfelf, fhe was rigidly enjoined to defift, as foon as the ufual effects of the

 medicine medicine appeared, until ſhe heard from either of us; but, as too frequently happens, our injunctions were diſregarded. The tincture was of my own preparing.

In a variety of caſes of the moſt acute and painful ſuffering, which ſhall, I truſt, in due time be ſubmitted to public inſpection, I have ventured to preſcribe 30, 35, and at one time, 40 drops at a doſe; the two former repeated twice a day, and very generally with the happieſt effects. In ſome inſtances, the habit has, in a few minutes, felt its ſalutary influence; and in general, the ſtomach, pulſe, and head, have been affected in twenty-four and forty-eight hours. In urgent caſes, as relief muſt be ſpeedy, ſo the doſe muſt be large, and proportioned to the urgency of ſymptoms.

In formidable caſes of meaſles, when the urgency of the cough, the quick, difficult, anxious, or laborious reſpiration, with a high fever, denote a dangerous diſeaſe; eſpecially if from the hiſtory of the family there be reaſon to apprehend a ſcrophulous or phthiſical diatheſis, but in which blood-letting, or the exhibition of opium, may be thought equivocal or hazardous, I beg, in a particular manner, to direct the attention of medical men to full doſes of the Digitalis. In ſuch caſes, fever is allayed, expectoration and all the other ſecretions promoted, reſpiration relieved, and the bowels relaxed, by its means, whereas the very reverſe is frequently the conſequence of opium. Hence its ſuperiority in many inſtances.

In the firſt caſe of meaſles in which I preſcribed it, I had every reaſon to attribute the ſafety of the patient entirely to its uſe. The ſame perſon, Mr. E. Oxley, of this town, was relieved in a few minutes, and permanently cured, by full doſes of it, of a moſt violent hemicrania, attended with intenſe heat and thirſt, with a feeble pulſe which beat about 140 ſtrokes in a minute. Laudanum in large quantities, and other means, were adminiſtered with evident aggravation of every ſymptom before I ſaw him. Being in the country when ſent for, they "thought," to uſe their own words, "he would go from his ſenſes before I arrived."

In a moſt violent caſe of external and internal hæmorrhoidal affection, now under my care, 40 drops gave almoſt immediate relief, and the patient is recovering rapidly by 35, evening and morning. When he firſt came to me, his countenance was pale and ſallow, his ſtrength and fleſh much exhauſted; he walked with extreme pain and difficulty; his pulſe was quick and ſmall, and his appetite impaired. In a week the contraſt was very ſtriking.

The diſeaſe had reſiſted judicious treatment for five weeks.

But in many other inſtances, as well as in conſumption, the practitioner practitioner muſt be prepared for diſappointments, by this, as by every other human means. Certain diſeaſes are beyond the reach of our art; and this may probably be wiſely intended by the Author of our being, in order to impreſs our minds with our own inſignificance. If the tinctures be properly prepared, of the genuine leaf, they will not be pale as brandy, but of a darker tinge than laudanum, of which two vials now before me exhibit a ſtriking proof.

I have this year dried, and am now drying, ſeveral pounds of the leaves of my own garden, on a net properly ſuſpended in a chamber through which a current of air paſſes, but from which the rays of the ſun are excluded; and, eſpecially when large quantities are prepared, this method, as being ſimple, eaſy, and certain, is perhaps preferable to any yet adopted.

Moſt cheerfully do I acknowledge myſelf indebted to Dr. Drake, and the celebrated author of the Zoonomia, for directing the attention of medical men more immediately to the tincture of Fox-glove. By means of this preparation I have been enabled to alleviate human miſery to a much greater extent than would otherwiſe have fallen to my lot to do. I can now readily furniſh a number of diſtreſſed objects, who come daily to implore my aſſiſtance, with a ſmall vial; whereas I had no means of providing them with the infuſion, or pills made of the powder. In this way, it is one of the higheſt gratifications of my life to reflect, that I have done much good, and, I truſt, may be enabled to do much more. When a decoction, or ſtrong infuſion is deemed neceſſary as an external application, my garden immediately ſupplies them.

An intereſting caſe in point, now comes to my recollection; —— Bridget, an infant a year old, was brought for my advice the 21ſt of November laſt. A highly inflamed ſcaly eruption, diſcharging a thin ichor, with here and there painful pimples, all over the head, had haraſſed it from its birth. Medicine had been reſorted to in vain. I gathered a handful or two of freſh leaves, with which I directed a ſtrong decoction to be made, and the head to be bathed with a little, evening and morning. It was brought to me again the 27th, nearly well; and on the 2d of December free of complaint. It gave me pleaſure to obſerve, ſoon after this, in Dr. Ferriar's excellent treatiſe, ſimilar happy reſults. In a caſe of true *tinea capitis* it failed with me; but in itch it ſeems an effectual remedy, of which the firſt hint was given me by Mr. Anderſon, an intelligent practitioner of this place.

The principles on which I give the preference to the tincture, in certain caſes, have been explained, (*ib. vol. iii. p.* 152, *et ſeq.*) and are very different from thoſe on which Dr.

Drake prefers it. He has thought proper to condemn the powder as a preparation, "by which the wifhed-for effect in confumption would feldom be produced," without trying it in a fingle inftance! This extraordinary affertion, without the fhadow of proof, I have already called to his recollection, (*ibid.*) and he has not attempted to contradict it; nor can he contradict it, without impeaching his own veracity; for he expreffly tells us, the tincture was the only preparation ufed in all the cafes he has brought before the public. By what authority then, or on what grounds, does he prefume to pronounce his anathema againft a fubftance he has never put to the teft of a trial in confumption? A fubftance too which has been ufed with fuccefs by others in the difeafe; and which has, under the eye of the higheft medical authorities in this kingdom, produced every effect on the human body of which the tincture is capable.—A word or two may, probably, tend to unravel this myftery.

In one of the cafes alluded to, in his communication for Oct. laft, (*ib. vol. ii. p.* 267) he tells us, "that the firft, a young man about twenty-eight years of age, had been under the care of two phyficians in the neighbourhood previous to his being called in." After drawing the hiftory of the cafe, I doubt not, very correctly, the reader is informed in a note thefe phyficians were "Dr. M'Lean and Dr. Clubbe, by whom the Digitalis had been adminiftered in the form of powder; the wifhed-for effect, however", continues he, "can feldom be produced by the powder, &c." "The tincture was pufhed to 120 drops in twenty-four hours." But after being three weeks under "its full influence, with the fymptoms nearly ftationary, he became averfe towards profecuting it farther; nor could I perfuade either him or his friends to permit a more extenfive trial. He died in five or fix weeks after." What inferences are intended to be deduced from thefe premifes? They appear to me obviouflly thefe:

1. That the powder having been adminiftered by two phyficians, had a fair trial; but,

2. That as the "wifhed-for effect is feldom produced by the powder," it failed as a natural confequence.

3. That had the tincture been given by thefe phyficians, fuccefs would moft probably have attended it, as general fuccefs was at this time promifed from it.

4. That had the tincture been perfevered in during the Doctor's pleafure, a happy refult would moft likely have been the confequence; for the failure is not attributed to want of efficacy of the medicine, but want of refolution of the patient, "the fymptoms being nearly ftationary." And,

Laftly,

Laftly, That thefe two phyficians fell under the heavy imputation of neglecting the only means of faving their patient.

I anticipate very different conclufions, however, from the candid reader, on perufing the following brief ftatement.

My firft vifit to Mr. Boutelle, of Polftead, the fubject of this cafe, I find by my notes to have been on the 24th of March, 1799. In addition to the fymptoms detailed by Dr. D. he was copioufly expectorating blood, generally florid, which he had done more or lefs from the commencement of his diforder. I faw him again on the 30th, and I perceive by my memorandum, I thought him fomewhat better. The Digitalis was now prefcribed, as follows:

℞. Fol. dig. p. pulv. gr. viij. G. arab. p. gr. vj. Confect. aromat. q. s. ft. Boli no. octo. fumt. 1 fing. noct. et matutinis.

Saline mucilaginous draughts were likewife prefcribed every fix hours.

The next tidings I had of him, from Mr. Salter the attending furgeon, were by letter a week after. I was informed that eight pills only were taken; that the laft two difordered his ftomach and head much; that he pofitively refufed perfevering in them any longer, as he thought himfelf worfe the whole time of taking them; that his friends and himfelf became reftlefs and impatient, and wifhed to have further advice. I was much furprifed to find, from Mr. Salter's letter, that the hæmoptoe was evidently aggravated during the ufe of the medicine. Having occafion to meet Dr. Clubbe of Ipfwich, in confultation, the following day, the 9th of April, in the neighbourhood, we were defired to fee Mr. B. and it is but juftice to Dr. Clubbe's fagacity and difcernment, to declare, that he pronounced the cafe at once hopelefs. I own there appeared to me ftill fome ray of hope. The Digitalis was not again prefcribed; for, as hinted on a former occafion, Dr. C. had been extremely unfuccefsful with it. I faw the patient no more.

As we promifed nothing, fo we neither defired, nor were folicited, to repeat our vifits. At this time the miraculous cures that had been, and were to be, performed by the tincture, were loudly thundered forth from the trumpet of fame. Hadleigh was within a few miles; the pleafing found foon reached the ears of the patient and friends. They naturally fought relief where they were taught to believe they were certain of finding it; "for the tincture had not as yet failed in a fingle inftance." The Doctor was called in. What were his promifes may be eafily conjectured. Of what followed we are apprifed. On the above ftatement I fhall only offer one or two further remarks.

When a favorite remedy fails, it unfortunately happens that the the author generally finds fomething or other at hand to account for the failure. Every thing unfavorable to his purpofe is fcrupuloufly kept in the back ground, while every favorable circumftance is exhibited to view, as evidently happened in the prefent inftance. "The powder was adminiftered by two phyficians," that is, it had a full and a fair trial; but it was not a preparation fuited to the cafe; hence, "the wifhed-for effect could not be produced." But had the tincture been at firft ufed, from which, on the Doctor's authority, general fuccefs was promifed, or had it been perfevered in afterwards, for "the fymptoms were nearly ftationary," the reader is left to infer that the cafe would have terminated otherwife than it did. The conftitution of this patient, as I have fince more than once experienced, was in a particular manner fufceptible of the noxious effects of the Fox-glove. Small dofes difordered every organ and function, and even encreafed the flow of blood from the veffels of the lungs. I firmly believe then, that it was one of thofe unfortunate cafes which would have refifted the falutary powers of the tincture of Fox-glove, and every other human aid, although judicioufly adminiftered from the firft week of his illnefs.

If the Doctor has condemned what he has not ufed, it is not furprifing that he fhould have difapproved of what he has not feen. His objections to, and mode of arguing againft, what I have faid on the culture of the Fox-glove in my garden, are of a moft peculiar caft. I affirmed, (*ib.* p. 121.) that in confequence of the difappointments experienced from the bad quality of the leaf the firft few years I prefcribed it, "I had for fome years cultivated the plant in my garden, whereby I had reafon to believe its virtues were improved; that I was certain they were not impaired." To which the Doctor thus replies, "With regard to the plant itfelf, I can affure Dr. M. he is deceived in fuppofing it to acquire greater vigour from cultivation in a garden. Nineteen times out of twenty he will find the reverfe to be the event. The Digitalis delights in an elevated, a light and gravelly foil; on a *fat* and *denfe* mould, in *low* and *damp* fituations, and where *ftrata* of *chalk* abound, it always degenerates, becomes dwarfifh, and of a paler green. Dr. M's houfe at Sudbury, is certainly fituated low, not far from the river, in the immediate neighbourhood of chalk-beds; on fuch a foil, it is evident none of the habitudes of the plant can be gratified; and by the ufual modes of improving a garden, the ground will be rendered ftill lefs fit for promoting its growth and ftrength, &c." (*Ib. vol.* ii. p. 421.)

I own I was not aware of the fhadow of an objection to what I ventured to fay on this head. I fancied to myfelf the facts above above ſtated, had been aſcertained both by my medical friends and me, after repeated trials, beyond the poſſibility of doubt. But Dr. Drake ſeems to poſſeſs the happy talent of acquiring that knowledge by intuition, which others cannot attain by experiment and attentive obſervation. For, as far as I know, he has never been within the walls of my garden; he has never ſeen the Digitalis growing in it; nor has he put the virtues of the leaf, thus reared, to the teſt of a ſingle experiment!

The reader will perceive that I ſpoke only of my *own* garden; and had he *poſitively* contradicted my aſſertion, I could have anſwered and confuted him in *one* word. Aware however, I preſume, of the conſequences of one gentleman calling in queſtion the veracity of another, without ſubſtantial evidence, he prudently avoids this, and, with a ſpecies of ſophiſtry that may impoſe on weak and ſuperficial minds, but too ſhallow, I truſt, not to be at once detected by men of diſcernment, ſhifts the ground, and fabricates a ſoil and ſituation for my garden, which exiſt only in his own imagination; and with an air of profound ſagacity tells us, the Digitalis will not thrive in ſuch a ſoil or ſituation. The terms "on a fat and denſe mould, in low and damp ſituations," &c. are not to be found in my remarks; and ſuch a ſoil and ſituation are certainly not thoſe of my garden. The gardeners and farmers of this country would laugh at his definition of the ſoil of our gardens and fields, and bluntly tell him he was totally ignorant of what he claims a familiar acquaintance with. They would aſſure him, they are neither chalky nor calcareous, nor denſe nor damp; that wherever chalk-beds are found, the chalk is ſeveral feet, in general many yards, beneath the ſurface, a thick ſolid ſtratum of gravel intervening; that neither the plough nor the ſpade ever reach it, except where the natural ſoil has been removed to fill up inequalities, as I have obſerved in a field in the pariſh of Henny, in Eſſex, a few miles from Sudbury; that although the ſoil may vie in fertility with any in the kingdom, it is rather of a looſe than of a denſe and ſolid texture. This is the ſum of the information I have received from the gardeners and farmers, and accords with my own obſervations; and few have traverſed its ſurface, for ten years, more frequently. But, for argument's ſake, admitting the ſoil, and every other circumſtance, to be as the Doctor repreſents them, does he imagine that any one, finding the Fox-glove growing luxuriantly in an elevated ſituation, in a light gravelly ſoil, would be ſo conſummate a blockhead as to plant it in "a fat, denſe mould, in a low, damp ſituation, &c.?" I can aſſure him we have no ſuch in this part of Suffolk. Our gardeners here have more intelligence than he ſeems aware of; they would inform him,

ſtrenuous

ſtrenuous as he is for leaving this plant in the hands of nature, that both this, and hundreds of others, originally growing in the fields, are now reared in gardens, in much higher luxuriance than in their original ſtate; that although ſome vegetables degenerate and die, yet many others are improved in all their properties by ſuch removal; that one of the principal ends of their art is, to improve upon Nature when ſhe is deficient, to imitate her when ſhe has attained the higheſt degree of perfection of which ſhe is capable.

Whoever has attended to the Digitalis in its natural ſtate, muſt have ſeen, that in the ſame field or hedge-row, ſome plants are in full health and vigour, while others are weak and dwarfiſh. Nature often diſtributes her gifts, for wiſe and ſalutary purpoſes, with a liberal, often with a ſparing hand; and the intelligent botaniſt, even the moſt ignorant gardener, well knows that the ſoil and ſituation in which ſhe has placed her numerous offspring, animals as well as vegetables, are not always the moſt congenial to them. But, far be it from my wiſh to adviſe the officious interference of art, where ſhe is bountiful of her gifts. It was not until I had experienced repeated diſappointments, that I was induced to cultivate this valuable plant within my immediate reach, and ſtrenuouſly to recommend it to many of my medical friends; and from the additional confidence which farther experience inſpires, I not only venture to repeat, what I have already ſaid in reply to the Doctor's objections, (*ib. vol. iii. p.* 151, *et seq.*) but affirm, that I never ſaw it in any place, and I believe it never did, attain to ſuch beauty and perfection, as well as height. I have heard of its growing ſix and ſeven feet high in its natural ſtate. I never ſaw this: The imagination of thoſe who argue like Dr. D. may readily magnify it to this height, while it would reduce that of my garden to dwarfs,—to mere pigmies. Dr. Withering ſays, " the ſtems are about four feet high." I have, I think, ſeen it higher than this: the talleſt of my produce, by accurate meaſurement lately, were five feet ten inches high; but thoſe of three and four feet throw out the ſtrongeſt and coarſeſt leaves. They have one peculiarity likewiſe, which I have not obſerved in the wild plant, which adds much to their beauty and elegance, and which they ſeem to derive from artificial culture. They have from four to ſix ſmaller ſtems, ſome of which are two feet in length, with ſeveral bloſſoms iſſuing from the principal ſtem, at twelve and eighteen inches from the ground. Theſe come later, and the bloſſoms continue longer than the principal ones: they are not in Withering's plate, or in any I have ſeen. Are they uſual?

The ſtems of the white plants are more delicate and ſlender; the the leaves of a paler green, of a ſmoother texture, and in general not ſo large as thoſe of the purple. I have diſcovered no difference, however, in their virtues. The two plants form a moſt beautiful contraſt; and as there is a ſucceſſion of bloſſoms for ſome weeks, few contribute more to the ornament of the flower garden.

Were it neceſſary to adduce farther facts, in oppoſition to general aſſertion, I might add, that two pariſhes (Higham and Lawford) adjoining to Eaſt Bergholt, in which the Doctor loaded his cart with Fox-glove, ſupplied me moſt liberally laſt ſummer, on which many trials were made.

What I have ſaid of the neceſſity of free expoſure to the ſun and air, &c. is not only ſtrictly correct, but a general law in regard to vegetables, with as few exceptions, at leaſt, as any other. When gathering the plants at Lawford Hall, on the grounds of my much reſpected friend, E. Green, Eſq. of that place, hundreds were rejected, becauſe they were ſo ſhaded by trees, and ſo ſurrounded by weeds and other plants, as, from want of ſun, air, and nutriment from the earth, not to arrive at a quarter of their natural ſize. They were thin, pale, and ſickly; ſome of the ſtems were tall, but very weak and ſlender. In a long and painful attendance lately on my truly worthy friend, the late Rev. Dr. Preſton, of this country, a ſtriking inſtance of this occurred to me: In his beautiful ſhrubbery I had often admired the luxuriant growth of the Fox-glove; of late, from cauſes unneceſſary to ſtate here, the grounds had been neglected, and were conſequently over-run with weeds; in vain did I for ſome time look for my old acquaintance; at length, however, I found a few weakly plants concealed by the weeds, without any *ſtems or bloſſoms.*

[ To be continued in our next. ]

